# An integrative taxonomic characterization of extremophile fish *A* *phanius almiriensis* (Teleostei: Cyprinodontiformes) in a hypersaline geothermal system (NW Anatolia)

**DOI:** 10.1111/jfb.70520

**Published:** 2026-05-30

**Authors:** Sevan Ağdamar, Gülşah Saç, Deniz Anıl Odabaşı, Müfit Özuluğ

**Affiliations:** ^1^ Department of Forestry, Bayramiç Vocational School Çanakkale Onsekiz Mart University Çanakkale Türkiye; ^2^ Department of Biology, Faculty of Science İstanbul University İstanbul Türkiye; ^3^ Department of Marine and Inland Water Sciences, Faculty of Marine Science and Technology Çanakkale Onsekiz Mart University Çanakkale Türkiye; ^4^ Natural Wealth Research and Application Center İstanbul University İstanbul Türkiye

**Keywords:** *Aphanius*, extreme conditions, killifish, mtDNA, restricted habitat

## Abstract

The small killifish *Aphanius almiriensis* (Kottelat, Barbieri & Stoumboudi, 2007) inhabits from brackish springs to estuaries and coastal lagoons in the eastern Mediterranean basin. This study focused on the detailed characterization of *Aphanius almiriensis* from a hypersaline geothermal spring (Tuzla, Çanakkale, NW Anatolia), where extreme salinity (61.14 ppt in winter, January 2023) and temperature (42.7°C in summer, July 2023) conditions were observed. The obtained specimens of killifish in the sampling site, with a slightly convex dorsal head profile and a more angular ventral head profile, are similar in this respect to specimens from Greece. In most individuals (25 out of 27), the two frontal scales do not contact each other. Genetic analyses indicate that the species exhibits a minimum p‐distance of 3.1% from *Aphanius fasciatus* in the mitochondrial cytochrome c oxidase subunit I (COI) region. Although previously reported, this population had not been thoroughly characterized up to date; however, based on the present study, it exhibits genetic and morphological characteristics consistent with *A. almiriensis* specimens from Greece.

## 1 INTRODUCTION

Following molecular studies in recent years, the definition, limits, and contents of the genus *Aphanius*, previously included in the family Cyprinodontidae, have been changed from a large genus to a much more restricted one (Esmaeili et al., 2020; Freyhof & Yoğurtçuoğlu, 2020). The family Aphaniidae currently comprises eight monophyletic genera: *Aphaniops*, *Anatolichthys*, *Apricaphanius*, *Esmaeilius*, *Kosswigichthys*, *Paraphanius*, *Tellia*, and *Aphanius*, of which the latter is represented by only two species, *Aphanius fasciatus* and *Aphanius almiriensis*, distributed in the Mediterranean basin, Iran, the Red Sea, and the Persian Gulf (Freyhof & Yoğurtçuoğlu, 2020; Triantafyllidis et al., 2007; Valdesalici et al., 2019). The Tethyan relict members of the genus inhabit the area with a rather surprising biogeographic pattern, inhabiting fresh and brackish or hypersaline water springs/coastal streams, lagoons, tidal channels, swamps, salt marshes, and estuaries (Freyhof & Yoğurtçuoğlu, 2020; Hrbek & Meyer, 2003; Triantafyllidis et al., 2007; Valdesalici et al., 2019).

Extreme water salinity or temperature conditions limit the presence of a variety of fish in water bodies (Eaton et al., 1995; Wang & Guo, 2019). Fishes in the order Cyprinodontiformes (commonly known as killifishes), comprising more than 1400 species, are a group of fishes that are well adapted to these extreme conditions and have a worldwide distribution across tropical and temperate regions, inhabiting a wide range of freshwater to hypersaline waters, including estuarine environments and desert springs (Akbarzadeh et al., 2014; Chiozzi et al., 2018; Piller et al., 2022).

Because *A. almiriensis* and *A. fasciatus* share numerous overlapping phenotypic traits, distinguishing between them in natural habitats proves to be a considerable challenge during fieldwork (Freyhof & Yoğurtçuoğlu, 2020). However, *A. almiriensis* was described as a new species from the Peloponnese (Greece) by Kottelat et al. (2007), immediately after Triantafyllidis et al. (2007) found two well‐separated molecular groups in *A. fasciatus* from Greece (Valdesalici et al., 2019). It has recently been recorded from a small coastal cenote in Italy, outside its known range; however, Valdesalici et al. (2019) suggested a probable allochthonous origin for this population, potentially dating back to Roman times. As reported by Triantafyllidis et al. (2007), *A. almiriensis* exhibits a broader distribution within the Aegean Basin and has recently been recorded along the coastal regions of Türkiye (Freyhof & Yoğurtçuoğlu, 2020).

*Aphanius almiriensis* was recently reported from Tuzla, NW Anatolia (Freyhof & Yoğurtçuoğlu, 2020), but without any detailed morphological or genetic characterization. Here, we provide the detailed morphological description and molecular identification of *A. almiriensis* (the population of Tuzla), thereby adding new insights into this species' variation under extreme environmental conditions. The objective of the present study is to identify the *Aphanius almiriensis* population inhabiting the Tuzla hypersaline geothermal spring at the species level using both morphological and molecular analyses.

## 2 MATERIALS AND METHODS

### 2.1 Study area and sampling

Çanakkale–Tuzla geothermal field, situated approximately 80 km southwest of Çanakkale and 5 km inland from the Aegean coast, is characterized as a volcanic region (Baba et al., 2009; Erenoğlu et al., 2019). The thermal waters, with a surface discharge temperature range of 34–80°C, originate from two distinct reservoirs: a shallow volcanic reservoir located at a depth of 330–350 m, and a deeper granitic reservoir at approximately 1020 m. The thermal waters of Tuzla are distinguished by their exceptionally high dissolved salt concentration, reaching up to 68 ppt, and their enriched content of calcium (Ca), sodium (Na), potassium (K), strontium (Sr), and chloride (Cl), surpassing those of other thermal water regions in Western Anatolia (Baba et al., 2009; Karamanderesi, 1986; Şimşek, 1997; Vengosh et al., 2002).

**FIGURE 1:**
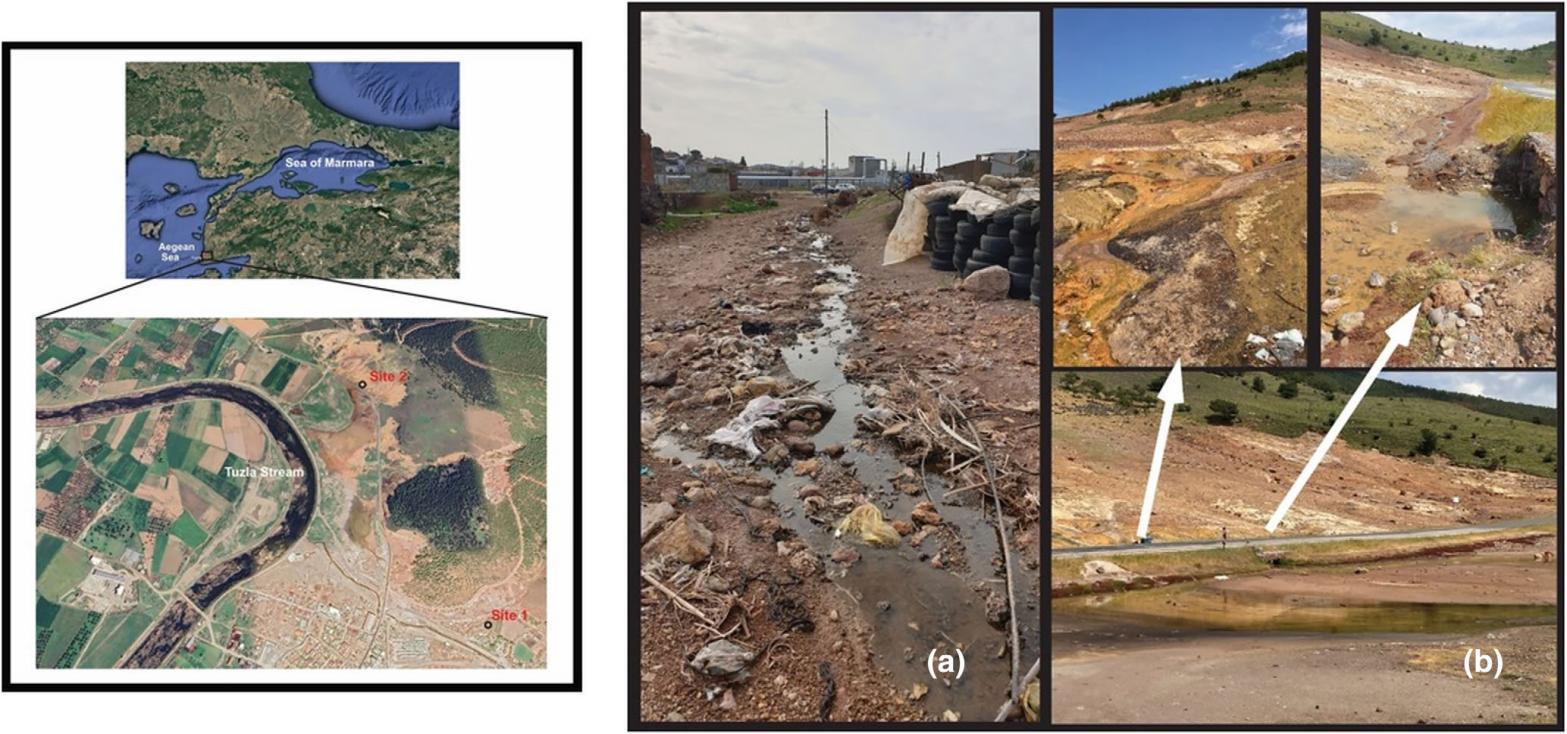
Map of the study area (Tuzla hypersaline geothermal spring, NW Anatolia) and sampling sites, and images of the study area (a: Site 1, b: Site 2).

Tuzla village located in the southwest of the Biga Peninsula (Çanakkale, Türkiye) is a region of thermal water outlets. On the tectonic fault line at the foot of a mountain near the village, geothermal evaporation causes the spring water to reach a temperature of around 70°C. Two independent springs have been observed in the region, and they are characterized as hypersaline geothermal waters, which are formed by a combination of hot and cold water sources. These springs, which flow separately through narrow channels, can reach the Tuzla Stream, which flows into the northern Aegean Sea. Although the springs flow throughout the year, their volume is reduced by evaporation during the summer months. The study was carried out in these two hypersaline geothermal springs (Figure 1):

*Site 1* (39.569036° N, 26.172661° E) was in the Tuzla village. It is a shallow (10–30 cm depth) hypersaline geothermal spring flowing through a narrow canal (30–40 cm width). This hypersaline geothermal spring was fed both by low‐flow water at a temperature of about 70°C, which was discharged by geothermal evaporation, and by cold leakage water in the rock.

*Site 2* (39.576950° N, 26.169161° E) was located 1 km north of the Tuzla village on the road. This hypersaline geothermal spring was also fed both by low‐flow water at a temperature of about 70°C and by cold leakage water in the rock.

The physico‐chemical parameters of the springs were measured using a multi‐parameter probe (Eutech CyberScan 600).

The springs, consisting of a mixture of hot and cold water flowing through rocks, were very shallow and had a relatively low flow rate compared to typical streams (Figure 1). The salinity and temperature of these hypersaline geothermal springs were measured at high levels, with 61.14 ppt in winter (January 2023) and 42.7°C in summer (July 2023) (Table 1). Notably, both salinity and temperature values exceeded those of seawater (approximately 38 ppt in the Mediterranean Sea), indicating that the fish tolerate extreme environmental conditions.

**TABLE 1:** Physico‐chemical parameters measured in the study area (Çanakkale, Türkiye).

Stations	Seasons	WT (°C)	pH	Salinity (ppt)	EC (μS/cm)	DO (mg/L)
Site 1	Winter	20.2	7.48	61.14	77.82	9.41
	Spring	28.2	6.95	61.09	88.19	7.38
	Summer	31.5	6.55	60.58	87.80	6.20
	Autumn	27.3	6.82	59.21	80.34	8.92
Site 2	Winter	23.7	6.99	60.7	76.3	8.94
	Spring	37.1	6.95	59.8	79.9	7.19
	Summer	42.7	6.62	55.2	75.0	5.51
	Autumn	40.0	6.81	47.7	63.9	7.49

Abbreviations: DO, dissolved oxygen; EC, electrical conductivity; WT, water temperature.

### 2.2 Material examined

The treatment of collected fish specimens was consistent with the Republic of Türkiye's animal welfare laws and guidelines, and the experiments were conducted with the permission of Çanakkale Onsekiz Mart University Committee of Animal Experiments Local Ethics (Decision ID: 2022/05‐03).

The study area was visited seasonally between January and October 2023, and 27 fish specimens were collected from the defined sampling sites using a scoop net with a mesh size of 3 mm. The collected specimens were sacrificed with an overdose of clove oil (100 mg/L). For morphometric measurements and meristic counts, some specimens were fixed in 4% formaldehyde solution and then stored in 70% ethanol. Additionally, caudal fin tissues of 10 specimens were directly preserved in 99% ethanol for subsequent molecular analysis.

The historical Tuzla specimens (collected in 1993) and additional materials from the Küçükçekmece and Büyükçekmece lagoons were included solely for comparative morphological evaluations across different time periods and localities. No molecular data were available for these historical and additional specimens.

#### 2.2.1 List of material collected from Tuzla thermal spring (Türkiye, Çanakkale prov)

IUSHM 2024‐1471, 14 ind., 27–32 mm SL; April 2023.

IUSHM 2024‐1472, 17 ind., 24–28 mm SL; January 2023.

IUSHM 2024‐1473, 14 ind., 19–40 mm SL; July 2023.

IUSHM 2024‐1474, 10 ind., 24–32 mm SL; August 1993.

IUSHM 2024‐1477, 18 ind., 14–26 mm SL; near to thermal bath in Tuzla village; January 2023.

#### 2.2.2 Additional material

IUSHM 2024‐1475, 12 ind., 35–48 mm SL; Türkiye, İstanbul prov., Küçükçekmece Lagoon; June 1973.

IUSHM 2024‐1476, 2 ind., 54–63 mm SL; Türkiye, İstanbul prov., Büyükçekmece Lagoon; June 1981.

### 2.3 Morphological analyses

Kottelat and Freyhof (2007) were followed for measurements and counts. All measurements were made with a digital calliper point‐to‐point and recorded to the nearest 0.1 mm. Standard length (SL) was measured from the tip of the snout to the end of the hypural complex. The length of the caudal peduncle was measured from behind the base of the posterior anal‐fin ray to the end of the hypural complex, at mid‐height of the caudal‐fin base. Lateral line scales were counted from the anteriormost scale (the first one to touch the shoulder girdle) to the last scale at the end of the hypural complex. Scales on the caudal fin itself are indicated by “+”. For comparisons, morphological data for *A. almiriensis* inhabiting Greece and Italy based on Kottelat et al. (2007) and Valdesalici et al. (2019) were used.

### 2.4 DNA extraction, PCR, and sequencing

Genomic DNA was extracted from caudal fin tissues using Qiagen DNeasy Blood & Tissue Kit. Cytochrome c oxidase subunit I (COI) gene region (654 base pairs) was amplified on tissue samples obtained from 10 specimens morphologically assigned to *A. almiriensis*, to assess genetic similarity among specimens and to support our morphological identification. COI target‐specific primer pairs (COI‐F: 5′‐TCAACCAACCACAAAGACATTGGCAC‐3′ and COI‐R: 5′‐TAGACTTCTGGGTGGCCAAAGAATCA‐3′) were designed to amplify only the DNA of target species from tissue content. The specificity of primer pairs was tested in silico using the ecoPCR (Dejean et al., 2011) and primerBLAST (Jerde et al., 2011) software. DNA amplifications were performed in 25 μL volumes, each containing: 2.5 μL of 10X Taq Buffer with KCl (100 mM Tris–HCl, 500 mM KCl, pH 8.8), 2.5 μL of MgCl_2_ (25 mM), 0.5 μL of dNTPs (10 mM), 0.5 μL of each primer (10 pM/μL), 1 U of Taq polymerase (5 U/μL), and 1 μL of DNA (50 ng/μL). PCR amplifications were performed in Eppendorf Mastercycler® with the following cycling conditions (modified from Ivanova et al., 2007): preliminary denaturation at 95°C for 5 min followed by 35 cycles consisting of denaturation at 95°C for 1 min, primer annealing at 54°C for 1 min, primer extension at 72°C for 1 min and final extension step at 72°C for 10 min. Sanger sequencing was performed by BM Labosis Ltd. Co. (Ankara, Turkey) with reverse primer (COI‐R).

### 2.5 Molecular data analyses

Mitochondrial DNA (mtDNA) sequences were aligned using the ClustalW algorithm in MEGA X (Kumar et al., 2018). The 10 COI sequences generated in this study were submitted to GenBank and the accession numbers PP218677‐PP218686 were assigned. Sequence similarity relationships were explored using a Neighbour‐Joining (NJ) tree based on uncorrected p‐distances with 1000 bootstrap replicates, as implemented in MEGA X (Kumar et al., 2018). Genetic distances between species were calculated in MEGA X (Kumar et al., 2018) using the p‐distance model with 1000 bootstrap replications. Reference sequences of the species on the NJ Tree were retrieved from GenBank (see Table S1).

## 3 RESULTS

### 3.1 Description

**FIGURE 2:**
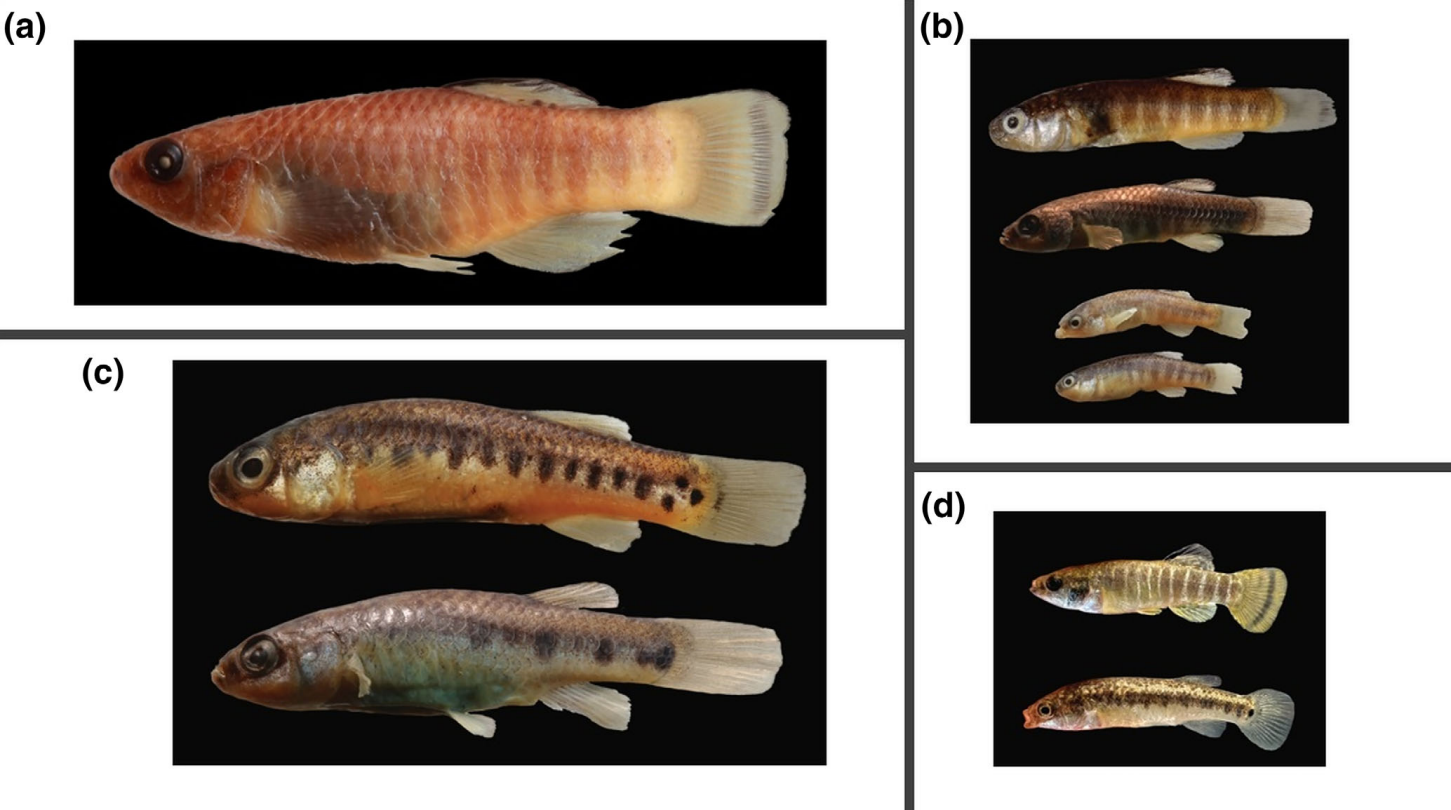
Composite images of *Aphanius almiriensis* specimens: (a) IUSHM 2024–1473, (in life) 25 mm SL male (top), 24 mm SL female (bottom) from Tuzla hypersaline geothermal spring (Çanakkale, Türkiye); (b) IUSHM 2024–1473, four male specimens, sorted from top to bottom by size (25, 23, 18, and 18 mm SL), from Tuzla hypersaline geothermal spring; (c) IUSHM 2024–1473, 30 mm SL (top), 27 mm SL (bottom) female, from Tuzla hypersaline geothermal spring; (d) IUSHM 2024–1475, 38 mm SL male from Küçükçekmece Lagoon.

**FIGURE 3:**
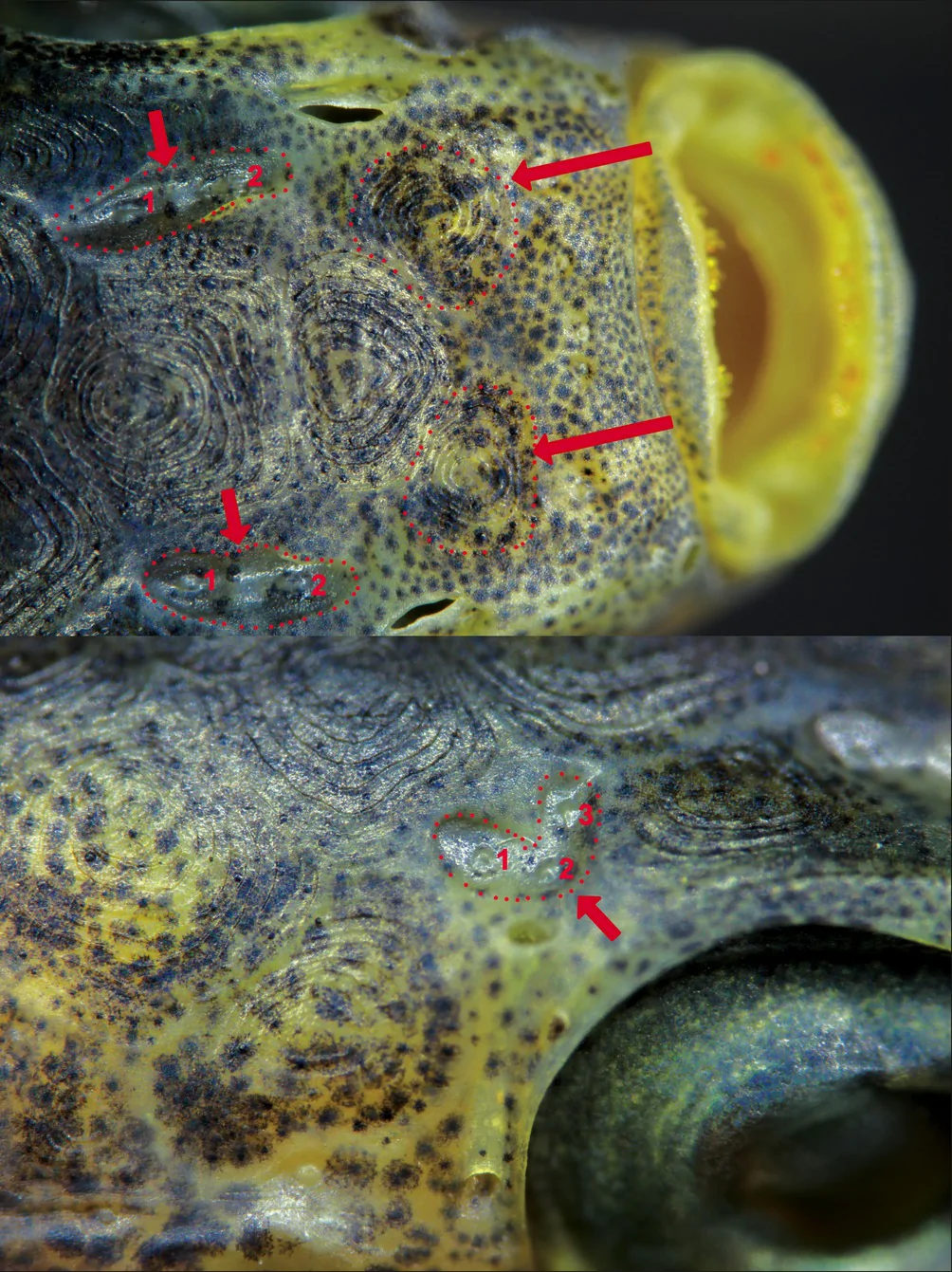
Two scales in the anterior frontal area and supraorbital groove with two neuromasts (top); postorbital curved groove with three neuromasts (bottom).

See Figure 2 for the general appearance of the species and Table 2 for the morphometric data of *A. almiriensis*. The lower jaw is slightly curved upwards. Teeth are tricuspid, with the median cusp longer than the lateral ones. The dorsal profile is slightly convex from the nape to the origin of the dorsal fin, while the ventral profile is less convex than the dorsal profile. The deepest point of the body is located between the pectoral and pelvic fins. The pectoral fin is rounded, with its posterior margin reaching the origin of the pelvic fin. The pelvic fin does not reach the origin of the anal fin. The caudal peduncle is laterally compressed. The origin of the anal fin lies below the vertical through the 3rd or 4th branched dorsal‐fin ray in both sexes. The dorsal and anal fins are rounded and do not reach the base of the caudal fin. The caudal fin is rounded or subtruncate. The dorsal and anal fins have 7½ (1), 8½ (9), or 9½ (5) branched rays. The caudal fin has 7 + 7 (1), 7 + 8 (2), 8 + 7 (3), or 8 + 8 (9) branched rays. The pectoral fin has 14 (10) or 15 (5) rays, and the pelvic fin has 6 (2) to 7 (13) rays. The trunk and head are covered with large, cycloid scales. The flank has 24 (2), 26 (11), or 27 (2) scales along the lateral series. Two or three additional rows of small scales are present at the anterior base of the caudal fin. There are 3–4 rows of scales between the dorsal fin and the lateral series, and 5–6 between the lateral series and the pelvic fin. The caudal peduncle bears 15 (1) to 16 (14) circumpeduncular scales. The peritoneum is black. Cephalic neuromasts are present in the interorbital area, located within a deep open groove. In most individuals (25 out of 27), the two anteriormost frontal scales do not contact each other (Figure 3). Metric observations of the Tuzla specimens revealed that the distance between the pelvic and anal fins differs between sexes; in males, the anal fin originates more anteriorly, resulting in a longer caudal peduncle, though this difference was not tested for statistical significance. The following colouration patterns are presented descriptively, as their diagnostic value relative to *A. fasciatus* remains to be fully established:

**TABLE 2:** Morphometric data of *Aphanius almiriensis* populations in Tuzla hypersaline geothermal spring area (Çanakkale, Türkiye).

Metric characteristics	*n*: 17 Female				*n*: 10 Male			
	Mean	Min	Max	±SD	Mean	Min	Max	±SD
Standard length (mm)	29.6	25.7	40.4	3.9	23.8	19.7	28.4	2.8
**In percent of standard length**								
Head length	30.7	29.2	31.9	0.8	29.6	28.2	32.8	1.4
Body depth at dorsal‐fin origin	22.9	21.0	24.4	1.1	22.0	20.1	24.9	1.6
Body width at dorsal‐fin origin	10.8	8.4	13.7	1.5	9.5	7.2	13.4	1.8
Predorsal length	65.2	63.7	66.9	0.9	63.8	62.0	68.1	2.0
Postdorsal length	24.8	22.2	27.1	1.4	27.2	24.2	30.0	1.7
Prepelvic length	50.8	49.5	52.5	0.9	49.6	48.0	53.1	1.5
Preanal length	68.9	65.6	71.3	1.5	65.3	64.3	67.5	1.0
Distance between pectoral and pelvic fin origin	22.6	21.4	25.1	0.9	20.2	18.5	24.6	1.7
Distance between pelvic and anal fin origin	19.0	17.1	20.8	1.1	16.8	15.9	18.2	0.8
Depth of caudal peduncle	14.4	12.8	15.9	0.7	14.9	13.5	17.2	1.1
Length of caudal peduncle	21.7	19.4	23.9	1.4	24.0	22.0	25.9	1.2
Pectoral fin length	19.2	17.2	20.8	1.0	20.8	18.9	22.5	1.1
Pelvic fin length	10.9	9.7	11.8	0.6	10.7	9.2	12.1	1.1
**In percent of head length**								
Head depth at posterior of preoperculum	64.3	59.7	72.5	2.9	65.7	63.0	69.0	2.1
Head depth at eye	53.2	49.8	58.8	2.6	54.6	49.6	59.9	3.3
Snout length	31.7	29.2	34.3	1.5	27.9	26.1	30.0	1.2
Eye diameter	31.0	24.5	34.0	2.6	31.3	29.3	33.8	1.6
Postorbital distance	45.4	43.5	48.1	1.3	45.6	43.7	47.6	1.4
Maximum head width	70.2	66.1	80.3	3.3	69.5	65,8	75.5	3.5
Interorbital width	39.0	36.5	45.0	2.1	37.3	34.3	41.0	2.5

#### 3.1.1 Preserved male colour

The upper part of the head is brownish, while the lower part of the head and the base of the pectoral fin are silvery. The lower cheek, breast, and belly are whitish. The dorsal portion of the head and the flank are brown. The flank displays 9–12 brown to dark‐grey bars. The first bar is located at the vertical of the pectoral‐fin base, and the last bar lies just anterior to the origin of the caudal fin. The pectoral and pelvic fins are hyaline. The anal fin is hyaline. The dorsal fin is hyaline with black pigmentation on the branched rays. A black dorsal‐fin margin is present, wide anteriorly and narrowing or absent on the last dorsal‐fin rays. The caudal fin is pale hyaline, with a dark vertical band present in some specimens.

#### 3.1.2 Preserved female colour

The upper part of the head is brownish‐grey. The lower part of the head and the base of the pectoral fin are whitish‐silvery. A series of 7–11 large blotches is present along the lateral midline, which are weakly elongated in a vertical direction in some individuals. Posterior to these spots, an irregular, faint, dark mid‐lateral stripe is present. A single blotch is found at the center of the caudal‐fin base in most individuals. In some individuals, two small, elongated, and irregularly shaped black blotches are present at the base of the caudal fin. All fins are hyaline.

### 3.2 Molecular data

**FIGURE 4:**
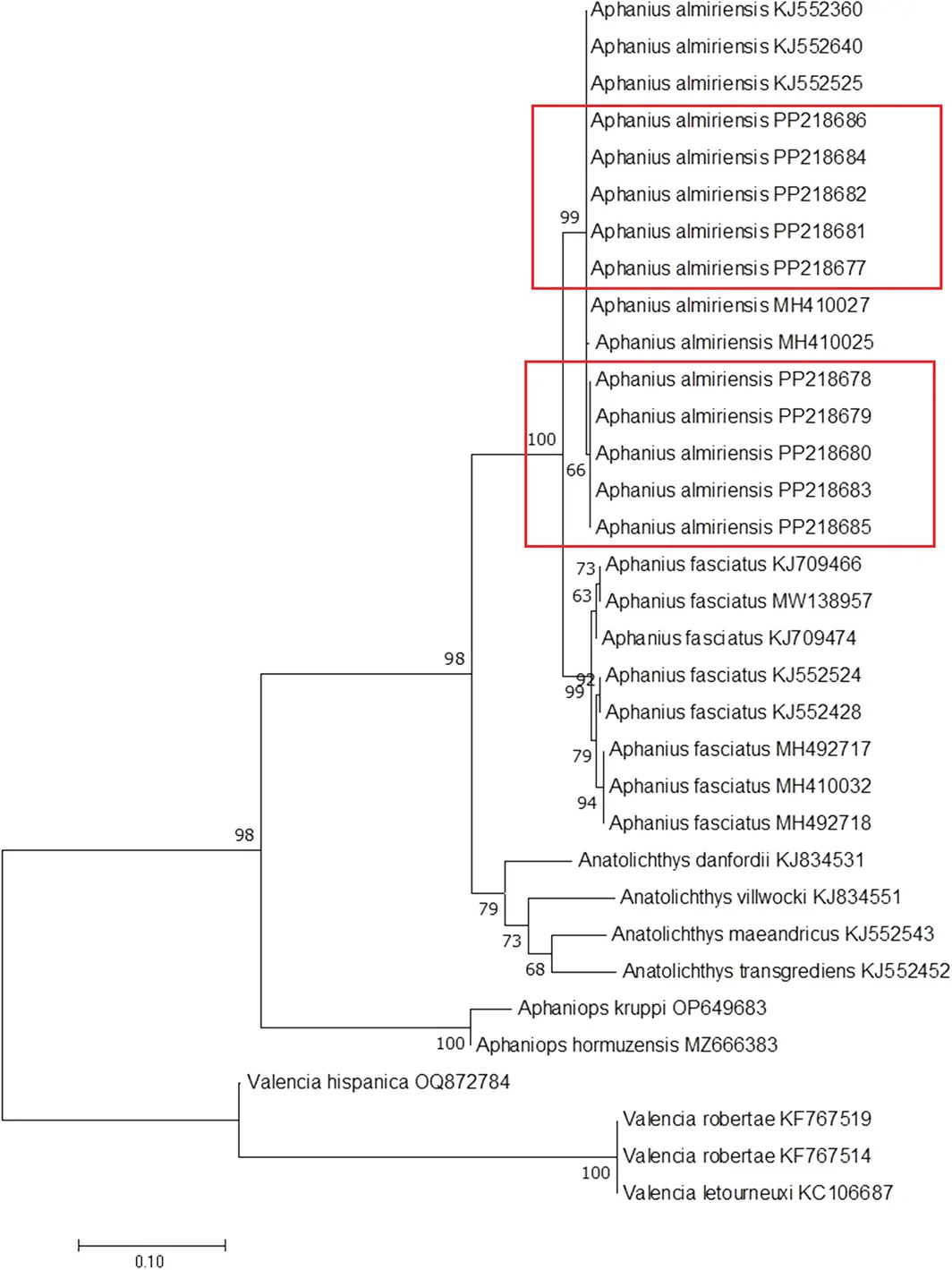
Neighbour‐Joining (NJ) tree based on the neighbour‐joining (NJ) analysis of pairwise genetic p‐distances. Bootstrap values for 1000 replicates are shown above branches on th e NJ tree (Scale: 1.0% p‐distance). The red‐squared sequence records pertain to the present study.

The morphological identification was subsequently explored by a neighbour‐joining (NJ) analysis reconstructed from mitochondrial cytochrome c oxidase subunit I (COI) gene sequences of 10 *A. almiriensis* specimens and their closest congeneric species, including sequences retrieved from GenBank (Figure 4). The COI sequences of the Tuzla population show haplotypic cohesion, sharing the same nucleotide sequence and clustering together (Figure 4), showing a minimum 3.1% genetic divergence from their closest relative, *A. fasciatus* (see Table S2). The NJ tree is consistent with our morphological identification, providing supportive exploratory evidence for the taxonomic classification of the studied species.

## 4 DISCUSSION

There is limited literature on *A. almiriensis*, which was first described from an open‐water spring at Almiri Beach in the Peloponnese and from the Meligou Lagoon in Greece (Kottelat et al., 2007). In this context, the present study provides a detailed morphological and molecular characterization of the population inhabiting the Tuzla hypersaline geothermal spring, thereby supporting its taxonomic identity and significantly extending its documented range. According to Kottelat et al. (2007), *A. almiriensis* is morphologically very similar to *A. fasciatus*, but differs from it with a stouter caudal peduncle, wider interorbital distance, and larger eyes. All species in Aphaniidae are morphologically homogeneous and have similar scale patterns, fin positions, and meristic characters (Freyhof & Yoğurtçuoğlu, 2020). Furthermore, as demonstrated by studies on Iranian freshwater and extremophile species, adaptation to extreme environments in Aphaniidae is not solely reliant on phenotypic and physiological changes (Teimori et al., 2022; Zarei et al., 2023). Unlike the brief description by Freyhof and Yoğurtçuoğlu (2020), this study reports morphological features and COI sequence data, offering a deeper understanding of the identity of this species. The present results support the assignment of the Tuzla population to *A. almiriensis*, extending the species' documented range with morphological and mtDNA data.

Kottelat et al. (2007) and Valdesalici et al. (2019) observed differences between *A. fasciatus* and *A. almiriensis* in the number and arrangement of neuromasts in the interorbital region and the configuration of frontal scales. *Aphanius almiriensis* has two neuromasts in a deep groove on each side of the interorbital area, and the two most anterior scales on the frontal area do not touch each other. However, one *A. almiriensis* individual was found to have a partially closed groove containing two neuromasts (Kottelat et al., 2007). In contrast, *A. fasciatus* possesses three neuromasts in three separate pores, and the two most anterior frontal scales are in contact. In another study, the first record of *A. almiriensis* from Italy was reported by Valdesalici et al. (2019); this population was characterized by two rostral neuromasts, 2–4 supraorbital neuromasts in a deep groove, and three postorbital neuromasts in a rounded, deep, open groove. In contrast, *A. fasciatus* has 2 + 4 rostral neuromasts, three supraorbital neuromasts in three pores, and 2–4 postorbital neuromasts in a curved groove (Valdesalici et al., 2019). In this study, the number and structure of the supraorbital neuromasts in the Tuzla specimens are found to be consistent with those of *A. almiriensis* populations from Italy and Greece, exhibiting 2–3 supraorbital neuromasts located within a deep groove. However, the number of postorbital neuromasts is typically 3–4, and they are located in a curved (ovoid in a few specimens), open, shallow groove. Only two specimens possess two postorbital neuromasts: in one specimen, both neuromasts are situated within a round groove, while in the other, they are positioned in a channel. The Tuzla specimens, therefore, differ from the *A. almiriensis* populations in Greece and Italy in both the number and the groove shape of the postorbital neuromasts (vs. 1–2 in a round groove in the Greek population and 3 in the Italian population). The neuromast structure observed in the Tuzla samples collected in 1993 was consistent with that of the current specimens. Following morphological examination of the Küçükçekmece and Büyükçekmece specimens, previously identified as *Aphanius fasciatus* (Meriç, 1986a, 1986b), it was determined that these specimens exhibited remarkable congruence with *A. almiriensis*, and their neuromast characteristics were similar in both number and shape to those of the Tuzla population. Both lagoon specimens were notably larger than those from Tuzla. Two individuals (one male and one female) from the Büyükçekmece Lagoon possessed two supraorbital neuromasts, although the deep groove was partially closed.

The neighbour‐joining (NJ) analysis of the mitochondrial sequence data yielded a consistent NJ tree with strong bootstrap support. In the molecular identification of *Aphanius* species through mtDNA sequences, it has been shown that COI marker effectively delineates species in accordance with traditional taxonomic classifications. COI data derived from *A. almiriensis* specimens are identical and form a distinct cluster, separate from those of the nearest *Aphanius* species.

## 5 CONCLUSION

Although the presence of *A. almiriensis* at the Tuzla hypersaline geothermal system was previously reported (Freyhof & Yoğurtçuoğlu, 2020), no detailed morphological or genetic characterization of the population was provided. In contrast, this study offers a descriptive dataset combining previously unavailable morphometric measurements and COI sequence data, thereby contributing new insights into the species' phenotypic and genetic profile. Given that Tuzla's hypersaline (max. 42.7°C and 61.14 ppt) waters constitute an extreme environment, the current study focuses substantially on morphological and molecular data in a taxonomic context. Despite living in water far more saline than typical habitats, the Tuzla population's meristic and neuromast characteristics fall within the known range of *A. almiriensis* from Italy and Greece. Despite the prolonged confinement to this extreme geothermal environment, the morphological traits of the studied population show notable consistency with other known populations. A detailed comparison of these morphological features across different *A. almiriensis* populations and the closely related *A. fasciatus* is summarized in Table 3.

**TABLE 3:** Morphological comparison between *Aphanius fasciatus* and distinct populations of *Aphanius almiriensis*.

Morphological feature	*Aphanius fasciatus*	*Aphanius almiriensis* (Greece) (Kottelat et al., 2007)	*Aphanius almiriensis* (Italy) (Valdesalici et al., 2019)	*Aphanius almiriensis* (Tuzla, Türkiye) (present study)
Caudal peduncle	Relatively slender (Kottelat et al., 2007)	Stouter	*Not specified*	Depth 1.3–1.8 times in length
Interorbital distance	Relatively narrow (Kottelat et al., 2007)	Wider	*Not specified*	34.3–45.0% HL
Eye size	Relatively small (Kottelat et al., 2007)	Larger	*Not specified*	24.5–34.0% HL
Frontal scales (two most anterior)	In contact (Kottelat et al., 2007)	Not in contact	*Not specified*	*Not in contact*
Rostral neuromasts	2 + 4 neuromasts (Valdesalici et al., 2019)	*Not specified*	2 neuromasts	*Not specified*
Supraorbital (interorbital) neuromasts	3 neuromasts in three separate pores (Kottelat et al., 2007)	2 neuromasts in a deep groove on each side of the interorbital area	2–4 neuromasts in a deep groove	2–3 neuromasts located within a deep groove (consistent with Italy and Greece populations)
Postorbital neuromasts	2–4 neuromasts in a curved groove (Valdesalici et al., 2019)	Not specified	3 neuromasts in a rounded, deep, open groove	3–4 neuromasts in a curved (ovoid in a few specimens), open, shallow groove

Although *A. almiriensis* is widely distributed in the Mediterranean basin from Italy to Türkiye, the water bodies where it has been identified are very limited. The species was assessed as being Least Concern (LC) following the IUCN criteria for the Red List. However, it is noted that current population trends are declining in the face of threats such as droughts, invasive/non‐native species, diseases, pollution, and natural system modifications (IUCN, 2025). The cyprinodontid fish *A. almiriensis* confronts heightened extinction risks attributable to its narrowly constrained habitat and critically low abundance, both of which jeopardize the maintenance of genetic diversity. Recently published monitoring data (Saç et al., 2025) from Site 2 highlight a disproportionately female‐skewed demographic structure within this population, a condition likely to intensify inbreeding depression and drive the population toward a genetic bottleneck. It is anticipated that comprehensive molecular and morphological studies in the basin, specifically in water bodies with extreme conditions like lagoons and saline‐thermal springs, will enhance current knowledge of its distribution and conservation status. In addition, further studies should focus on the genetic mechanisms of the species for these extreme conditions.

## AUTHOR CONTRIBUTIONS

Sevan Ağdamar: Substantial contribution in the concept and design of the study; contribution to field data collection; contribution to molecular and data analyses; contribution to manuscript preparation. Gülşah Saç: Contribution to field data collection and manuscript preparation. Deniz Anıl Odabaşı: Contribution to field data collection and manuscript preparation. Müfit Özuluğ: Contribution to field data collection; contribution to data analyses and manuscript preparation. All authors have read and agreed to the recent version of the manuscript.

## FUNDING INFORMATION

This study was supported by the Research Fund of Çanakkale Onsekiz Mart University (Project No: FHD‐2022‐4179).

## CONFLICT OF INTEREST STATEMENT

The authors declare no conflicts of interest.

## Supporting information


**Table S1.** List of newly generated and GenBank retrieved COI sequences of *Aphanius* species used in the molecular analysis of *A. almiriensis*.
**Table S2**. Genetic p‐distances based on mitochondrial sequences (COI) of the species used in this study.

## Data Availability

The datasets generated during and/or analysed during the current study are available from the corresponding author on reasonable request.
