# Supplementary material for: An integrative taxonomic characterization of extremophile fish A phanius almiriensis (Teleostei: Cyprinodontiformes) in a hypersaline geothermal system (NW Anatolia)
Source: J Fish Biol. 2026 May 30;109(2):1261–9. doi: 10.1111/jfb.70520 (PMC13521695; doi:10.1111/jfb.70520)
Supplement: Supplementary file 1 [file JFB-109-1261-s001.docx]

**TABLE S1** List of newly generated and GenBank retrieved COI sequences of *Aphanius* species used in the molecular analysis of *A. almiriensis.*

| **Species** | **Location** | **GenBank Acc. No.** | **Reference** |
| --- | --- | --- | --- |
| *Aphanius almiriensis* | Greece | KJ552360 | Geiger et al. 2014 |
| *Aphanius almiriensis* | Greece | KJ552640 | Geiger et al. 2014 |
| *Aphanius almiriensis* | Greece | KJ552525 | Geiger et al. 2014 |
| *Aphanius almiriensis* | Italy | MH410025 | Unpublished |
| *Aphanius almiriensis* | Italy | MH410027 | Unpublished |
| *Aphanius fasciatus* | Cyprus | MW138957 | Unpublished |
| *Aphanius fasciatus* | Italy | KJ709466 | Landi et al. 2014 |
| *Aphanius fasciatus* | Italy | KJ709474 | Landi et al. 2014 |
| *Aphanius fasciatus* | Tunisia | KJ552524 | Geiger et al. 2014 |
| *Aphanius fasciatus* | Tunisia | KJ552428 | Geiger et al. 2014 |
| *Aphanius fasciatus* | Italy | MH492717 | Unpublished |
| *Aphanius fasciatus* | Croatia | MH410032 | Unpublished |
| *Aphanius fasciatus* | Italy | MH492718 | Unpublished |
| *Anatolichthys danfordii* | Türkiye | KJ834531 | Pfleiderer et al. 2014 |
| *Anatolichthys villwocki* | Türkiye | KJ834551 | Pfleiderer et al. 2014 |
| *Anatolichthys maeandricus* | Türkiye | KJ552543 | Geiger et al. 2014 |
| *Anatolichthys transgrediens* | Türkiye | KJ552452 | Geiger et al. 2014 |
| *Aphaniops kruppi* | Oman | OP649683 | Zarei et al. 2023 |
| *Aphaniops hormuzensis* | Iran | MZ666383 | Teimori et al. 2022 |
| *Valencia hispanica* | Spain | OQ872784 | Unpublished |
| *Valencia robertae* | Greece | KF767514 | Freyhof et al. 2014 |
| *Valencia robertae* | Greece | KF767519 | Freyhof et al. 2014 |
| *Valencia letourneuxil* | Greece | KC106687 | Vogiatzi et al. 2014 |

**TABLE S2** Genetic p-distances based on mitochondrial sequences (COI) of the species used in this study.

|  | Species | 1 | 2 | 3 | 4 | 5 | 6 | 7 | 8 | 9 | 10 | 11 | 12 | 13 | 14 | 15 | 16 | 17 | 18 | 19 | 20 | 21 | 22 | 23 | 24 |
| --- | --- | --- | --- | --- | --- | --- | --- | --- | --- | --- | --- | --- | --- | --- | --- | --- | --- | --- | --- | --- | --- | --- | --- | --- | --- |
| 1 | *A. almiriensis* |  |  |  |  |  |  |  |  |  |  |  |  |  |  |  |  |  |  |  |  |  |  |  |  |
| 2 | *A. almiriensis* | 0.000 |  |  |  |  |  |  |  |  |  |  |  |  |  |  |  |  |  |  |  |  |  |  |  |
| 3 | *A. almiriensis* | 0.000 | 0.000 |  |  |  |  |  |  |  |  |  |  |  |  |  |  |  |  |  |  |  |  |  |  |
| 4 | *A. fasciatus* | 0.031 | 0.031 | 0.031 |  |  |  |  |  |  |  |  |  |  |  |  |  |  |  |  |  |  |  |  |  |
| 5 | *A. fasciatus* | 0.034 | 0.034 | 0.034 | 0.003 |  |  |  |  |  |  |  |  |  |  |  |  |  |  |  |  |  |  |  |  |
| 6 | *A. fasciatus* | 0.037 | 0.037 | 0.037 | 0.010 | 0.008 |  |  |  |  |  |  |  |  |  |  |  |  |  |  |  |  |  |  |  |
| 7 | *A. fasciatus* | 0.039 | 0.039 | 0.039 | 0.013 | 0.010 | 0.008 |  |  |  |  |  |  |  |  |  |  |  |  |  |  |  |  |  |  |
| 8 | *A. fasciatus* | 0.031 | 0.031 | 0.031 | 0.000 | 0.003 | 0.010 | 0.013 |  |  |  |  |  |  |  |  |  |  |  |  |  |  |  |  |  |
| 9 | *A. danfordii* | 0.102 | 0.102 | 0.102 | 0.112 | 0.110 | 0.107 | 0.112 | 0.112 |  |  |  |  |  |  |  |  |  |  |  |  |  |  |  |  |
| 10 | *A. maeandricus* | 0.120 | 0.120 | 0.120 | 0.120 | 0.117 | 0.125 | 0.123 | 0.120 | 0.097 |  |  |  |  |  |  |  |  |  |  |  |  |  |  |  |
| 11 | *A. transgrediens* | 0.117 | 0.117 | 0.117 | 0.131 | 0.128 | 0.125 | 0.123 | 0.131 | 0.070 | 0.068 |  |  |  |  |  |  |  |  |  |  |  |  |  |  |
| 12 | *A. villwocki* | 0.131 | 0.131 | 0.131 | 0.131 | 0.128 | 0.125 | 0.123 | 0.131 | 0.091 | 0.086 | 0.094 |  |  |  |  |  |  |  |  |  |  |  |  |  |
| 13 | *A. kruppi* | 0.183 | 0.183 | 0.183 | 0.175 | 0.178 | 0.175 | 0.180 | 0.175 | 0.191 | 0.198 | 0.191 | 0.196 |  |  |  |  |  |  |  |  |  |  |  |  |
| 14 | *A. hormuzensis* | 0.188 | 0.188 | 0.188 | 0.180 | 0.183 | 0.180 | 0.185 | 0.180 | 0.188 | 0.185 | 0.185 | 0.185 | 0.026 |  |  |  |  |  |  |  |  |  |  |  |
| 15 | *A. almiriensis* | 0.003 | 0.003 | 0.003 | 0.034 | 0.037 | 0.039 | 0.042 | 0.034 | 0.104 | 0.117 | 0.115 | 0.128 | 0.185 | 0.185 |  |  |  |  |  |  |  |  |  |  |
| 16 | *A. fasciatus* | 0.039 | 0.039 | 0.039 | 0.013 | 0.010 | 0.008 | 0.000 | 0.013 | 0.112 | 0.123 | 0.123 | 0.123 | 0.180 | 0.185 | 0.042 |  |  |  |  |  |  |  |  |  |
| 17 | *A. fasciatus* | 0.039 | 0.039 | 0.039 | 0.013 | 0.010 | 0.008 | 0.000 | 0.013 | 0.112 | 0.123 | 0.123 | 0.123 | 0.180 | 0.185 | 0.042 | 0.000 |  |  |  |  |  |  |  |  |
| 18 | *A. almiriensis* | 0.000 | 0.000 | 0.000 | 0.031 | 0.034 | 0.037 | 0.039 | 0.031 | 0.102 | 0.120 | 0.117 | 0.131 | 0.183 | 0.188 | 0.003 | 0.039 | 0.039 |  |  |  |  |  |  |  |
| 19 | *A. almiriensis* | 0.000 | 0.000 | 0.000 | 0.031 | 0.034 | 0.037 | 0.039 | 0.031 | 0.102 | 0.120 | 0.117 | 0.131 | 0.183 | 0.188 | 0.003 | 0.039 | 0.039 | 0.000 |  |  |  |  |  |  |
| 20 | *A. fasciatus* | 0.037 | 0.037 | 0.037 | 0.010 | 0.008 | 0.000 | 0.008 | 0.010 | 0.107 | 0.125 | 0.125 | 0.125 | 0.175 | 0.180 | 0.039 | 0.008 | 0.008 | 0.037 | 0.037 |  |  |  |  |  |
| 21 | *V. robertae* | 0.214 | 0.214 | 0.214 | 0.222 | 0.219 | 0.217 | 0.219 | 0.222 | 0.196 | 0.217 | 0.214 | 0.217 | 0.235 | 0.232 | 0.217 | 0.219 | 0.219 | 0.214 | 0.214 | 0.217 |  |  |  |  |
| 22 | *V. robertae* | 0.214 | 0.214 | 0.214 | 0.222 | 0.219 | 0.217 | 0.219 | 0.222 | 0.196 | 0.217 | 0.214 | 0.217 | 0.235 | 0.232 | 0.217 | 0.219 | 0.219 | 0.214 | 0.214 | 0.217 | 0.000 |  |  |  |
| 23 | *V. letourneuxi* | 0.214 | 0.214 | 0.214 | 0.222 | 0.219 | 0.217 | 0.219 | 0.222 | 0.198 | 0.219 | 0.217 | 0.219 | 0.235 | 0.232 | 0.217 | 0.219 | 0.219 | 0.214 | 0.214 | 0.217 | 0.003 | 0.003 |  |  |
| 24 | *V.hispanica* | 0.230 | 0.230 | 0.230 | 0.217 | 0.214 | 0.214 | 0.214 | 0.217 | 0.201 | 0.196 | 0.204 | 0.188 | 0.201 | 0.191 | 0.227 | 0.214 | 0.214 | 0.230 | 0.230 | 0.214 | 0.164 | 0.164 | 0.167 |  |
